# Isolation of *Pseudomonas syringae* pv. *Tomato* strains causing bacterial speck disease of tomato and marker-based monitoring for their virulence

**DOI:** 10.1007/s11033-023-08302-x

**Published:** 2023-04-19

**Authors:** Bahaa E. S. Abd El-Fatah, Muhammad Imran, Kamal A.M Abo-Elyousr, Amer F. Mahmoud

**Affiliations:** 1 Department of Genetics, Faculty of Agriculture, Assiut University, 71526 Assiut, Egypt; 2 Department of Arid Land Agriculture, King Abdulaziz University, 80208 Jeddah, Saudi Arabia; 3 Department of Plant pathology, Faculty of Agriculture, Assiut University, 71526 Assiut, Egypt

**Keywords:** Bacterial speck, PCR amplification, ITS1analysis, *hrpZ* gene, RFLP

## Abstract

**Background:**

The bacterial speck disease of tomato caused by a bacterial pathogen *Pseudomonas syringae* pv. *tomato* is a most important disease causing severe crop losses.

**Methods and results:**

Present study was conducted to investigate and characterize the population diversity of *P. syringae* pv. *tomato* pathogen isolated from infected tomato plants from various regions of Egypt. Significant variation among the isolates was observed which demonstrated considerable virulence. All isolates were pathogenic and the CFU population recovered from inoculate tomato leaves by isolate Pst-2 was higher than other isolates. Genetic disparity among the isolates was investigated by PCR analysis by amplifying *hrp*Z gene using random amplified polymorphic DNA (RAPD), sequence-related amplified polymorphism (SRAP), and inter-simple sequence repeats (ISSR) markers. The amplified products for ITS1 were found to have 810 bp length whereas 536 bp length was observed for *hrp*Z gene using primer pairs (1406-f/23S-r) and (MM5-F, MM5-R) respectively. The restriction analysis of amplified regions “ITS” and *hrpZ* by using 5 and 4 endonucleases respectively demonstrated slight variation among the bacterial isolates. The results of RAPD, ISSR and SRAP showed higher polymorphism (60.52%) within the isolates which may assist for successful characterization by unique and specific markers based on geographical distribution, origin and virulence intensity.

**Conclusion:**

The results of present study suggested that the use of molecular approach may provide successful and valuable information to differentiate and classify *P. syringae* pv. *tomato* strains in future for the detection and confirmation of pathogenicity.

**Supplementary Information:**

The online version contains supplementary material available at 10.1007/s11033-023-08302-x.

## Introduction

Tomato (*Solanum Lycopersicum* Mill.) is considered as a most important vegetable crop widely grown worldwide [1, 2]. Various biotic and abiotic factors influenced the production of tomato whereas, bacterial diseases results in the significant yield reduction. Various bacterial diseases of tomato has widely been reported due to enormous crop losses [3–5]. Among the bacterial diseases of tomato, bacterial speck disease caused by *Pseudomonas syringae* pv. *tomato* [6] is one of the most important and widespread disease-causing massive yield losses in severe conditions. However, precise identification and confirmation of virulence is an important aspect to monitor the disease eruption. Thus, PCR amplification by 16 S and internal transcribed spacer 1 (ITS1) region are most commonly used for this purpose. Internal transcribed spacer 1 (ITS1) region splits 16 S rRNA–23 S rRNA while ITS2 separates 23 S rRNA–5 S rRNA gene. Nonetheless, sequence analysis of ITS1 region of bacterial species represents the length and variation among the sequences [7]. Therefore, the occurrence of variation in ITS1 region not only represents the difference between strains but also illustrates their intercistronic level. Various studies has documented that absence/existence of tRNA gene and incorporation/removal of nucleotides confers the intra-genomic heterogeneity of ITS1 region [8, 9] and frequent deletion results in the length discrepancy of ITS1 [9, 10]. However, for the specific bacterial genotype, amplification of *hrp*Z gene for internal fragments can provide substantiation of that bacterial strain. Further, a chromosomal gene “*hrp*Z” exists in *hrc*/*hrp* pathogenicity domain of *P. syringae* pv. *tomato* and has achieved considerable importance due to the development of symptoms on host. But on non-host plant, these genes results in hypersensitive response [11]. The DNA sequence of *hrp*Z gene of *P. syringae*, amplified by PCR with specific primers demonstrated various deletions, base substitutions, insertions and subsequent alteration in amino acids [12]. Thus, this technique provides the modern basis of identification and characterization of bacterial population. However, considerable genetic diversity in the population of *P. syringae* has been widely reported with Polymerase chain reaction-Restriction fragment length polymorphism (PCR/RFLP), Sequence-related amplified polymorphism (SRAP), Inter simple sequence repeats (ISSR) and Random amplified polymorphic DNA (RAPD) [13, 14].

For the digestion of genomic DNA with restriction endonuclease enzymes, RFLP was discovered and extremely sensitive analysis towards diminutive changes of DNA group [13] were observed and larger genomic part were examined with multi-locus enzymes analysis and DNA segments without regarded to protein expression or coding capability. The application of RAPD approach was firstly reported by Williams [15] and to date, several researchers has reported the helpful use of RAPD markers at population level and the detection of genetic diversity in controlled trials [16–18]. Afterward, for the investigation of microsatellite repeats without DNA sequences, ISSR markers were introduced [19] and this approach was reliant on the amplification of DNA segments between two microsatellite repeats [20]. Moreover, this approach was efficient, and simple whereas, the length of amplified product was ranging from 200 to 2000 bp.

In SRAP system, promoter and GC-rich exons are used to amplify the Open Reading Frames (ORFs) [21], which not only amplify the intervals between non-coding flanking regions of genes but also cause the compactness of actual gene linkage, which might produce the fingerprint of coding sequences and allow the easy isolation and rapid sequencing of bands [22].

Keeping the importance of these approaches, the aim of the present investigation was to: Identify and characterize the isolates of *P. syringae* pv. *tomato* from tomato from different regions in Egypt and to perform pathogenicity investigations to study the level of virulence with specific PCR, RFLP along with molecular marker system. However, fewer studies has reported the use of these markers and to the author’s knowledge, this is the first time to report the bacterial speck disease observed in Egypt. Typical symptoms of bacterial speck disease on tomato plants were observed in fields with the presence of dark brown/black (turning yellow halo) lesions on leaflets which might also appears on mature/older leaves.

## Materials and methods

### Isolation of pathogen

For the isolation of pathogen, naturally infected tomato leaves showing typical bacterial speck disease symptoms were collected in winter season 2018 from different localities of Assiut and El-Minia Governorates of Egypt. Leaves were rinsed thrice with sterile distilled water and uniformly segmented. The dissected leaves were surface sterilized with sodium hypochlorite (2% solution) for 3–5 min followed by rinsing with sterilized distilled water thrice. Subsequently, leaf tissues were pulverized in 2 ml potassium phosphate buffer (0.05 M) followed by the incubation at room temperature for 10 min. Afterwards, the homogenate was streaked on nutrient agar NA (peptone 5 g; yeast extract 1.5 g; beef extract 1.5 g, sodium chloride 5 g; agar 15 g; distilled water 1000 ml) medium plates with a sterilized bacterial rod [23]. Plates were incubated at 27 ± 2 °C for 48 h and the germinated bacterial colonies were observed. Bacterial colonies were purified by transferring single colony culture to a new NA medium plate to gain the pure bacterial culture that were maintained at 4^o^C for in glass slants containing NA medium for further studies.

### Pathogenicity assay

#### Plant material

In order to assess the level of pathogenicity of bacterial isolates, seedlings of a commonly grown tomato variety “Super Strain B” were grown in 20 cm pots containing sand and peat moss (1:3). At 3–4 leaf stage, seedlings were transplanted to new identical pots that were placed in greenhouse of the Department of Agriculture, Assiut University, Egypt. To fulfill the nutrient requirements, slow release fertilizer NPK (12:4:6) was added into soil [24]. The optimal temperature (30 ± 2 °C) and relative humidity (68–80%) inside the greenhouse was maintained and plants were irrigated as per requirement.

### Inoculum preparation

To differentiate the level of pathogenicity of isolates, four week old tomato plants were inoculated with the bacterial suspension of isolates. In order to prepare the inoculum of bacterial isolates, a bacterial loop from 48 h old colony culture (priorly grown on NA medium) was suspended in 50ml flasks containing 25ml nutrient broth medium (prepared as NA, lacking agar) followed by an incubation at 27 ± 2 °C on a shaker (120 rpm). After that 72 h old culture was centrifuged at 6000 rpm for 20 min at 4 °C. Pallet was discarded and supernatant was transferred to another sterilized flask and number of bacterial cells in the suspension of each isolate was adjusted with a spectrophotometer (Optical density OD_610_). The resulting suspension was further diluted with sterilized distilled water and numbers of cells per milliliter (ml^− 1^) were maintained. Tomato plants were sprayed (30ml/plant) with the final adjusted bacterial suspension containing (5 × 10^8^ CFU ml^− 1^) and six replicates were used for each isolate whereas, four plants were subjected as a replicate. Plants were covered with sterilized polythene plastic for 48 h to initiate the disease infection and plants were monitored daily to study the symptoms development. Symptoms were observed after 14 days of inoculation and disease severity of pathogenicity was measured with a 1–3 disease rating scale as reported and disease severity (%) was calculated [25]. Koch’s postulated were followed by isolating the bacterial cultures from inoculated leaves and compared to the original isolates. Experiment was repeated twice to confirm the virulence level of all isolates.

### Determination of bacterial population in infected leaves

Effect of isolated bacterial strains on the number of colony forming units (CFU) in inoculated tomato leaves was determined and the recovered CFU were calculated. In order to determine CFU populations, various uniform size discs (5 mm) were excised from infected area of leaf and homogenized in 1ml sodium chloride (0.06%) solution. The supernatant was serially diluted and the aliquot (0.1 ml) from each dilution was spread over the surface of NA medium plates with a sterilized glass spreader. Plates were incubated at 27 °C for 48 h and germinated colonies were observed. Colonies were enumerated with a magnifier and hand counter (Tamaco LTD, Taichung Taiwan) in each plate. Three dilutions from each leaf disc were prepared and used to determine the colony forming units (CFU) population in inoculated tomato leaves. Six replicates were used for each isolate and nine infected leaves were used as a replicate to measure the number of CFU population.

### Identification of the pathogenic bacteria

Pathogenic bacterial strains were initially identified based on their cultural, morphological, and physiological characteristics and then, based on well-known characteristics, pathogenic bacterial strains demonstrating typical bacterial speck disease symptoms on tomato plants were identified according to the interpretations available in the Bergey’s Manual of Systematic Bacteriology [26]. Whereas bacterial isolates were identified by PCR analysis using specific primer pairs.

### Molecular identification

#### Extraction of genomic DNA and PCR amplification

Genomic DNA of all bacterial isolates was extracted as reported [27] and DNA concentration was observed by UV-Vis spectrophotometer (Thermo Fisher Scientific™) at 260 nm (optical density OD_260_) and DNA quality was observed with agarose gel (0.8%) followed by staining with 0.5 µg ethidium bromide solution for 10 min and visualized with an ALPHA gel imaging system.

Subsequently, standard PCR reaction was performed for the identification of *P. syringae* pv. *tomato* with a primer pair, 1406-F (TGC TAC ACA CCG CCC GT) and 23 S-R (GGG TTC GTC CCC ATT CGA G) whereas, targeted internal transcribed spacers (ITS1) region among 16 S rRNA and 23 S rRNA gene (Fisher and Triplett 1999) was amplified. A primer pair MM5F (GAA CGA GCT GAA GGA AGA CA) and MM5R (CAG CCT GGT TAG TCT GGT TA) was used to amplify *hrpZ-pst* gene [28].

### Amplification of ITS1 and *hrp*Z regions

To perform the PCR amplification of target regions, PCR reaction mixture was prepared with a total volume of 25µL containing: 3.0µL of 10X PCR-buffer, 3.0 µL of dNTPs, 1.0 µL of each primer, 4.0 µL of MgCl_2_ (25 mM), 0.25 µL of Taq DNA polymerase, 1 µL template DNA and deionized H_2_O to make the volume up to 25 µL. Standard PCR reaction was performed in a thermal cycler^™^ with following reaction conditions: Initial denaturation at 94 °C for 5 min followed by 40 cycles, 94 °C for 1 min, 65 °C for 1 min, 56 °C for 30s and final incubation at 72 °C for 10 min. The reaction was cooled at 4 °C for 10 min. For the primer pair MM5-F and MM5-R, annealing temperature was maintained at 57 °C for 1 min keeping without interruption of other reaction. PCR products were visualized on 2% agarose gel in 1× Tris-acetate (TAE) buffer followed by staining in 0.5 µg ethidium bromide solution for 10 min. The length of PCR products was visualized with an Alpha gel imaging system.

### Genetic diversity

#### Restriction fragment length polymorphism (RFLP)

Five endonucleases, TaqI, BamHI, HindIII, HaeIII, MspI (Fermentas GmbH, St. Leon-Rot, Germany) were used for RFLP analysis of ITS region of *P. syringae.* pv. *tomato* strains with the primer pair 1406-f and 23 S-r (Fisher and Triplett 1999) and four endonucleases (TaqI, HaeIII, HindIII and NeaI) were used for digestion of specific fragment from the *hrp*Z*pst* gene. Amplified DNA products were precipitated in 2.5 µl ethanol (96%) and 0.1 µl of NaAc (3 M) with the final pH 3.2. Products were further deliquesced in 11 µl of Tris acetate (TAE) buffer containing EDTA (1 mM), 10 mM TRIS with the final pH 8.0. Then, the DNA solution (1 µl) was analyzed to estimate DNA concentration in agarose gel (2%) electrophoresis. For restriction analysis with each selected enzyme, 2 µl DNA was used and RFLP products were visualized in 2% agarose gel.

#### SRAP, ISSR and RAPD detection

Amplification of genomic DNA with SRAP, ISSR, RAPD markers was performed with primers/specific primer pairs (Table 1). Briefly, genomic DNA extracted as previously described was used to perform standard PCR reaction. The PCR reaction mixture was performed with a final volume of 25 µL containing identical volume as previously described. Standard PCR reaction was performed in a thermal cycler™ Senso Quest. PCR reaction conditions for these three markers were used as previously reported [29]. The gel was stained with ethidium bromide (0.5 µg) solution for 10 min and Alpha gel Imager system was used to visualize the amplified products. For each isolate, the presence/absence of DNA amplicons against each primer/primer pair was observed and genetic resemblance between bacterial isolates was deliberated according to the reported description [30] and based on reported similarities a dendrogram was constructed with NTSYS-pc (version 2.11T) [31]. Primer parameter i.e., polymorphic information content (PIC) was calculated as: PIC = 1[(p)^2^ + (q)^2^] [32], marker index (MI) was calculated as: MI = PIC x ηβ [33] while resolving power (Rp) was calculated as: Rp = ∑Ib [34].

**Table 1:** List of RAPD, ISSR and SRAP primers used in this study

Primer			Sequence 5’→3’	Primer	Sequence 5’→3’	
*RAPD primers*				*ISSR primers*		
OPB18			CCACAGCAGT	UBC807	AGAGAGAGAGAGAGAGT	
OPA15			TTCCGAACCC	UBC809	AGAGAGAGAGAGAGAGG	
OPC02			GTGAGGCGTC	UBC827	TGTGTGTGTGTGTGTGA	
OPU19			GTCAGTGCGG	UBC811	GAGAGAGAGAGAGAGAC	
OPB06			TGCTCTGCCC	UBC818	CACACACACACACACAG	
OPW06			AGGCCCGATG	IS10	TCCTCCTCCTCCTCCAC	
***SRAP primers***						
	**Forward primer** Sequence 5’→3’			**Reverse primer** Sequence 5’→3’		
SRAP-1	Me1		GAGTCCAAACCGGATA	Em2	GACTGCGTACGAATTTGC	
SRAP-2	Me2		TGAGTCCAAACCGGAGC	Em3	GACTGCGTACGAATTGAC	
SRAP-3	Me5		TGAGTCCAAACCGGAAG	Em4	GACTGCGTACGAATTTGA	
SRAP-4	Me6		AGTCCAAACCGGTAG	Em5	GACTGCGTACGAATTAAC	
SRAP-5	Me10		TGAGTCCAAACCGGGAC	Em6	GACTGCGTACGAATTGCA	
SRAP-6	Me1		GAGTCCAAACCGGATA	Em4	GACTGCGTACGAATTTGA	
SRAP-7	Me6		AGTCCAAACCGGTAG	Em2	GACTGCGTACGAATTTGC	
SRAP-8	Me5		TGAGTCCAAACCGGAAG	Em3	GACTGCGTACGAATTGAC	

### Statistical analysis

The statistical analysis of all the recorded data was performed with statistix (version 8.1) and the means of all treatments were compared using least significant difference (LSD) test at P ≤ 0.05 [35].

## Results

### Isolation and pathogenicity assay

From the naturally infected tomato leaves, a total of five bacterial isolates were obtained that were purified and tested to assess the level of virulence and differentiate the pathogenicity on tomato plants. The results of pathogenicity analysis showed that all five isolates were pathogenic and considerably produce typical bacterial speck disease symptoms identical to the symptoms observed in field under natural infection conditions. However, significant variation among the pathogenicity of all isolates was recorded (Fig. 1a). The pathogenicity of isolate “Pst-2” was relatively higher than other isolates. Whereas, the isolate “Pst4” was also found highly pathogenic but the level of virulence was relatively lower than “Pst-2” followed by Pst-1, Pst-3 and Pst-5. The results of this study demonstrate that all the obtained isolates were pathogenic which confers the existence and prevalence of bacterial pathogen in tomato growing fields.

**Fig. 1a:**
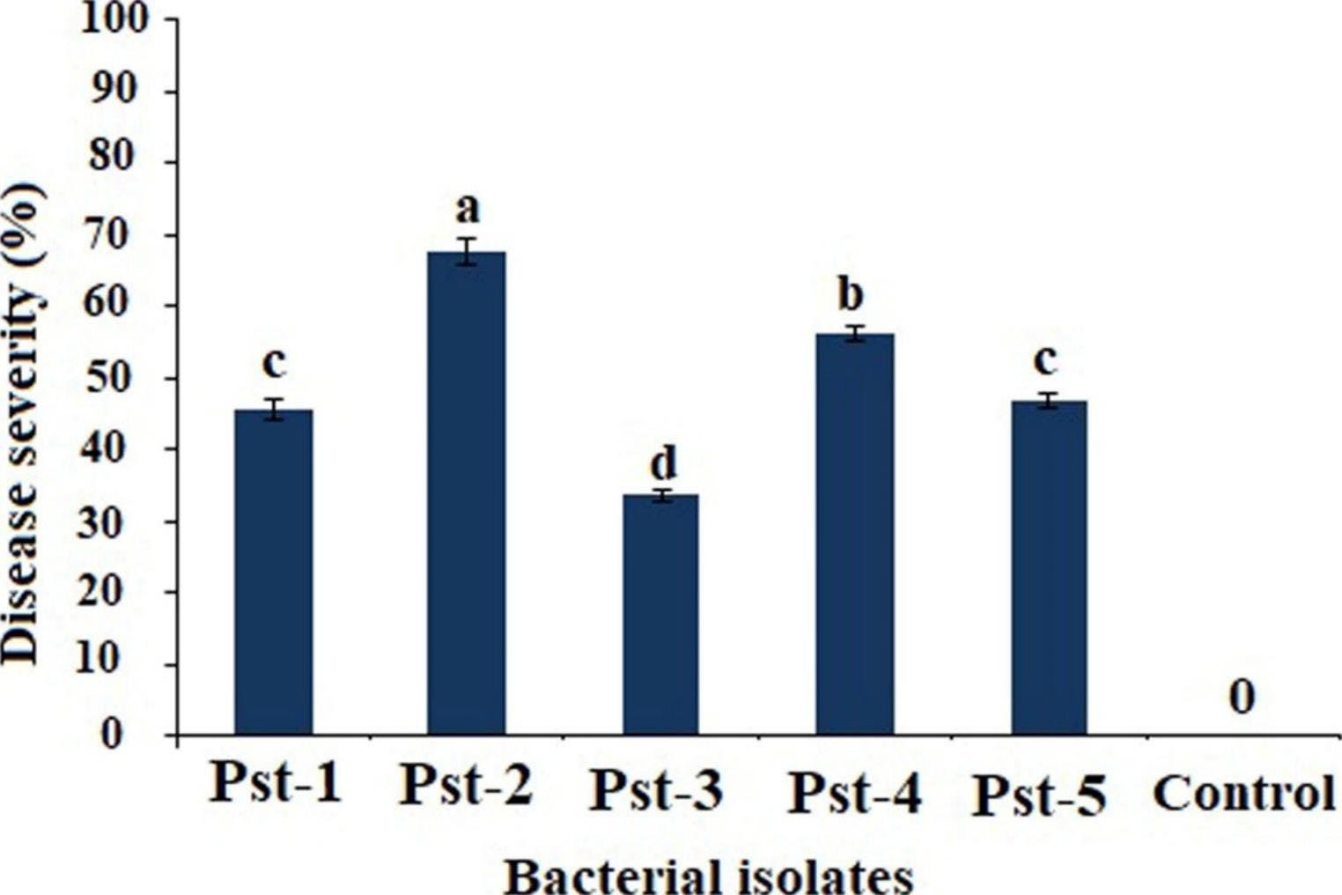
Pathogenicity and level of virulence of *Pseudomonas syringae* (Pst) isolates on tomato plants. The different letter indicates the difference among the isolates and bars indicate the mean ± SD at p ≤ 0.05 according to the fisher’s least significant difference (LSD) test

### Assessment of bacterial population in tomato leaves

The colony forming unit (CFU) population of *Pseudomonas syringae* in inoculated tomato leaves demonstrated significant variation among the CFU production by all bacterial isolates. As results, higher CFU population in the plants inoculated with isolate “Pst-2” was recorded followed by other isolates (Fig. 1b). Thus, the presence of CFU population and their considerable multiplication in treated plants confers the pathogenicity of all bacterial isolates. In these results, the bacterial isolate “Pst-2” showed higher CFU production than other isolates whereas, no significant (P ≤ 0.05) difference was recorded among the isolate “Pst-1” and “Pst-3” (Fig. 1b). Furthermore, the recovered CFU population from the infected tomato leaves clearly corroborates the pathogenicity of these bacterial isolates causing bacterial speck disease on tomato.

**Fig. 1b:**
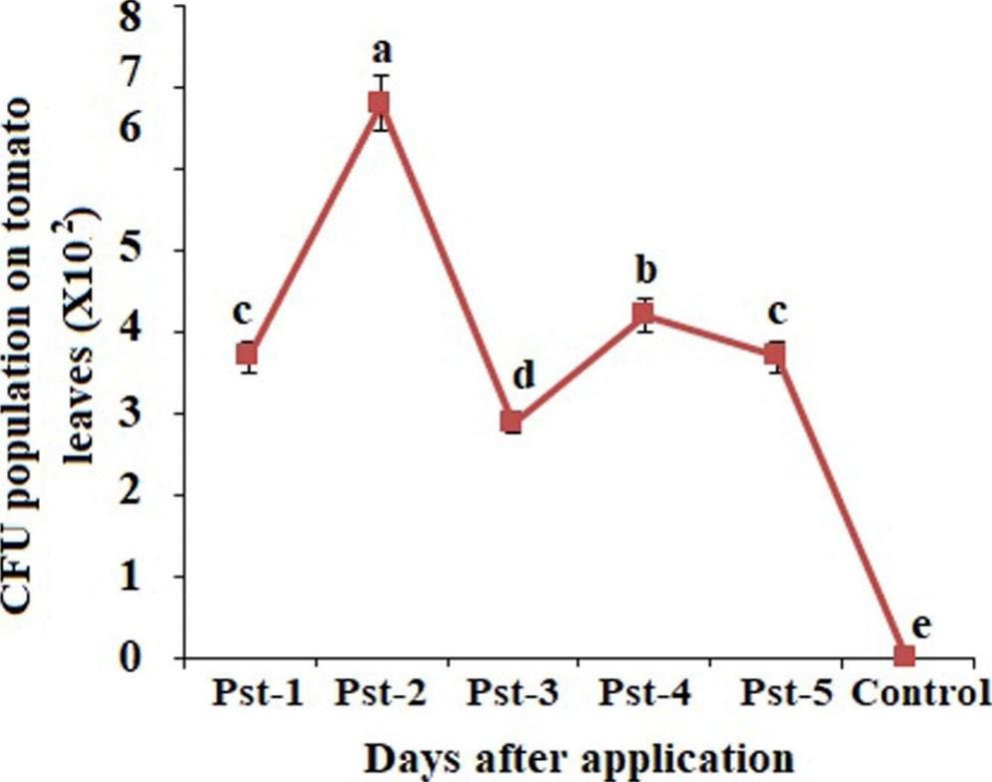
The Colony forming unit (CFU) population of *Pseudomonas syringae* bacterial strains in inoculated tomato leaves collected from greenhouse according to Zhang et al. 2022 [36]. The values followed by different letters indicates that means are significantly different (p ≤ 0.05) according to the fisher’s least significant difference (LSD) test and error bars demonstrated the mean ± SD of the replicates

### Morphological and molecular identification

**Fig. 2:**
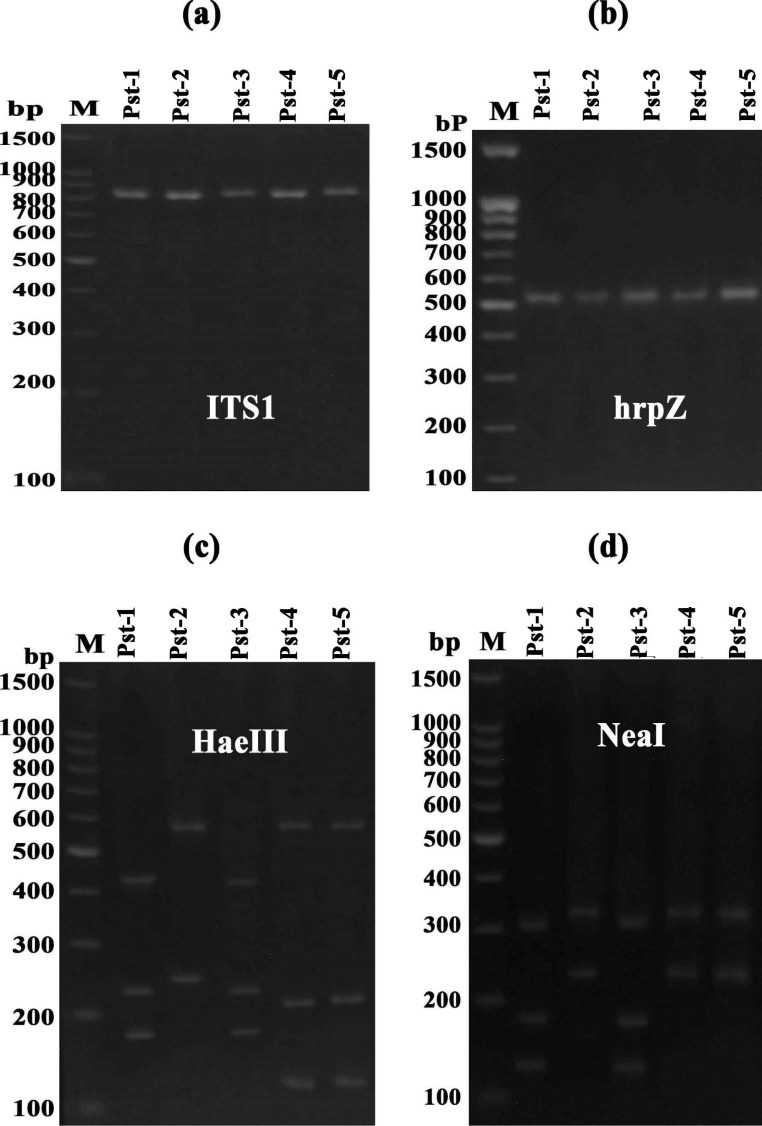
Gel electrophoresis analysis of PCR products amplified from *P. syringae* pv *tomato*. Reactions performed using different primers **(A)** ITS1 region analysis **(B)***HrpZ* gene amplification analysis **(C)** using Hae III endonucleases and **(D)** RFLP analysis using Nea I endonuclease. (M: Marker; Line 1–5 bacterial isolates)

Initially, the physiological and morphological characteristics viz., cell shape, motility levan production and fermentation of carbon compounds by pathogenic bacterial strains were studied. The rod shape of all bacterial isolates was observed (Supplementary Table S1). Further, all the bacterial strains were characterized as gram negative, motile and aerobic which were unable to produce the spore and pigmentation. The bacterial strains demonstrated significant levan production but were unable to produce sufficient indole. Bacterial isolates were found negative for starch hydrolysis test and potato rot but showed positivity for gelatin liquefactions. The results of present study demonstrate that all bacterial isolates considerably produce sufficient amount of lactose, glucose, fructose, sucrose and galactose acid. Whereas, the bacterial isolates were unable to produce raffinose acid, mannose and arabinose (Supplementary Table S1) In our results, the observed pathogenic, physiological, morphological and cultural characteristics were considerably identical to the previously reported characteristics [26] and thus, all the isolates were identified as *P. syringae* pv. *tomato.* Thus, for molecular identification, the primers pair 1406-f and 23-S amplified the specific ITS1 region of *P. syringae* pv. *tomato* between 16 S rRNA and 23 S rRNA genes with three bands of approximately 750, 800 and 900 bp length. However, the length of two amplified bands 750 and 900 bp was very weak and not reliable (Fig. 2a). Whereas, the primers MM5F and MM5R for *P. syringae* pv. *tomato* isolates amplify the expected pathovar-specific with 536 bp amplicons from the *hrpZ*pst gene of these five isolates **(**Fig. 2b).

### Genetic diversity

#### PCR-RFLP analysis

The amplification of ITS region by RFLP markers amplified the products at 800 bp length with five restriction enzymes (TaqI, BamHI, HindIII, HaeIII, MspI). However, the bacterial isolates were not differentiated by four endonucleases viz., TaqI, HindIII, BamHI and MspI. Additionally, by scoring the restriction fragments of HaeIII endonuclease, the ITS regions of *P. syringae* pv. *tomato* isolates were compared and the amplicon restriction in the isolate “Pst-2” with HaeIII assembled two fragments (240 and 570 bp). Whereas, three fragments were observed from the isolate “Pst-1” and “Pst-3” having 180 bp, 220 and 410 bp length for each and three fragments with the length of 30 bp, 210 and 570 bp for isolate “Pst-4” and “Pst-5” (Fig. 2c) were observed. Further, the *hrpZ* gene products obtained by RFLP analysis with three endonucleases enzymes (TaqI, HaeIII and HindIII) demonstrated no significant difference between the amplicon pattern of the isolates collected from Assiut and El-Minia Governorates.

The PCR products digested with endonuclease enzymes TaqI showed six cleavage sites at 34, 45, 50, 65,108, 234 bp, whereas the enzyme HaeIII produces four fragments of 30, 66, 114 and 326 bp molecular sizes. By using HindIII enzyme, the digestion of 536 bp fragment showed four fragments of 20, 32, 44 and 440 bp length while, the enzyme “NeaI” digested 536 bp fragment into two fragments of 216 and 320 bp length in three isolates (Pst-2, Pst-4 and Pst-5) that were collected from the different locations of Assiut Governorate. On the other hand, the isolates “Pst-1” and “Pst-3” showed the digestion of 536 bp fragment into three fragments having 36, 186 and 314 bp in length that were isolated from two locations of El-Minia Governorate (Fig. 2d).

#### RAPD, ISSR and SRAP

**Fig. 3:**
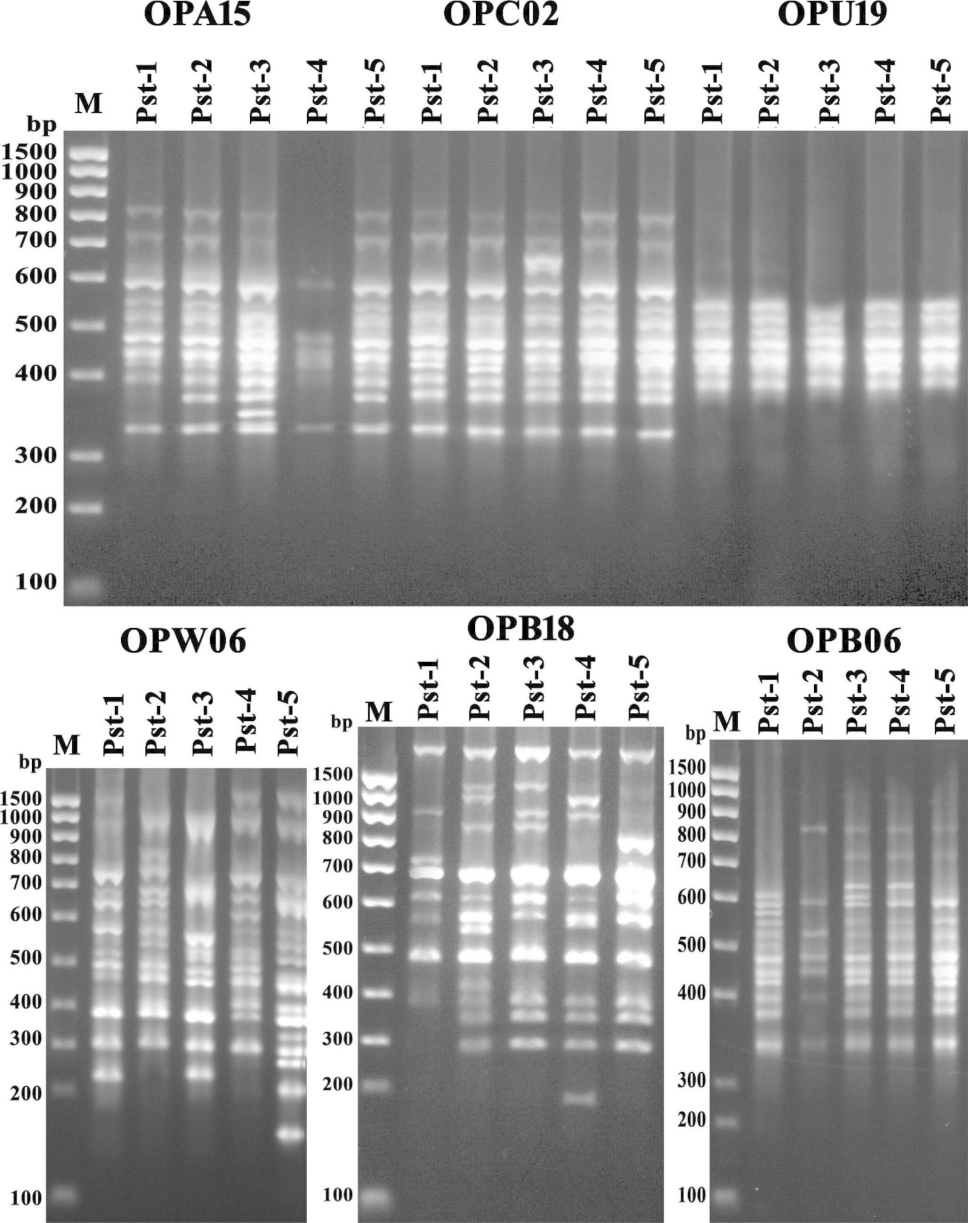
Gel stained with ethidium bromide showing PCR amplification products (bands) generated from the *P. syringae pv tomato* strains with RAPD (OPA, OPC, OPU) primers (M: Marker; Line 1–5 bacterial isolates)

**Fig. 4:**
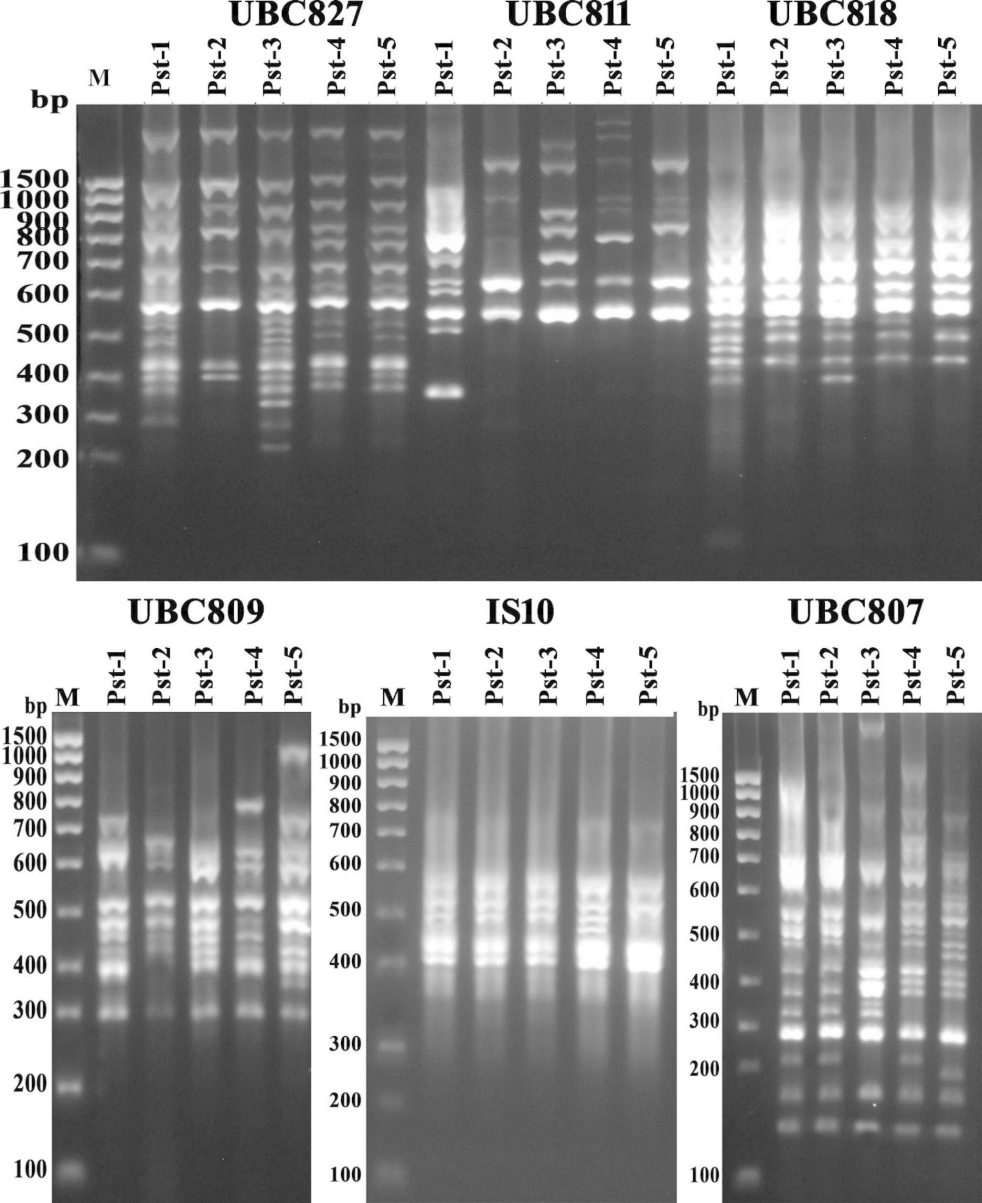
Gel stained with ethidium bromide showing PCR amplification products generated from the *P. syringae pv tomato* strains with ISSR (UBC, IS) primers (M: Marker; Line 1–5 bacterial isolates)

**Fig. 5:**
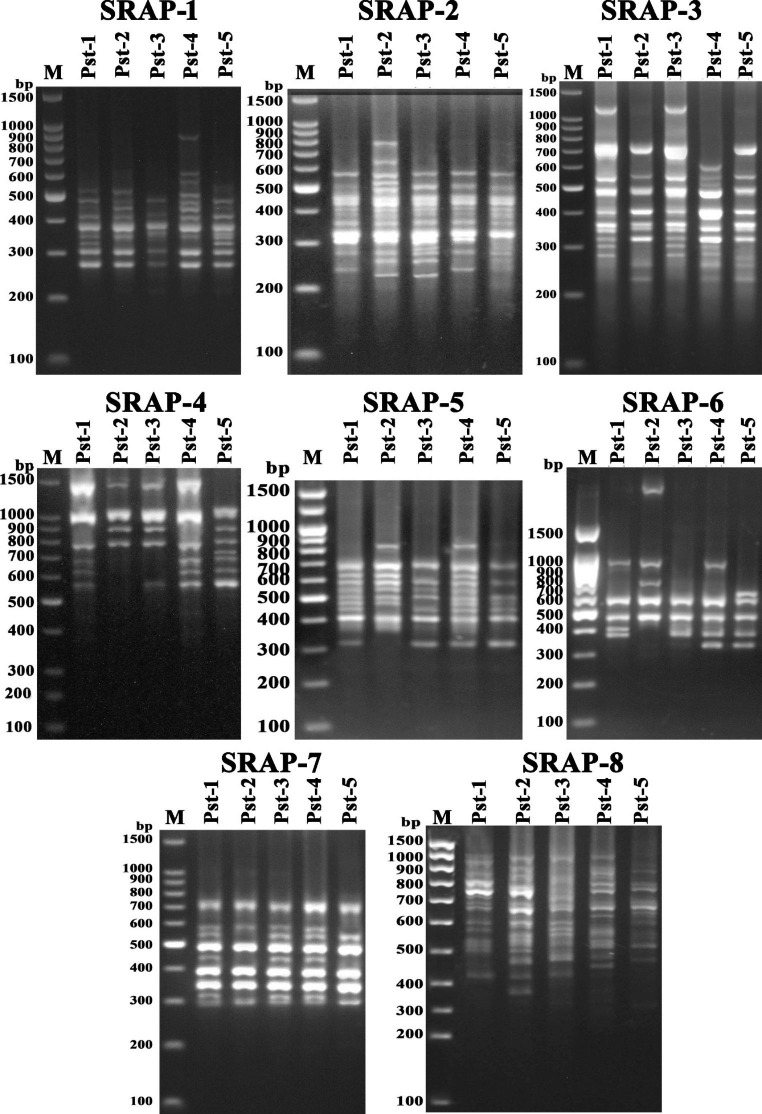
Gel stained with ethidium bromide showing PCR amplification products generated from the *P. syringae* pv *tomato* strains with SRAP primer combinations (M: Marker; Line 1–5 bacterial isolates)

The results of molecular characterization and differentiation in polymorphism of bacterial isolates using SRAP, ISSR and RAPD markers has been presented (Figs. 3, 4 and 5) that determine the molecular uniqueness of marker for each bacterial isolate. Using six RAPD primers, a total of eighty-four bands (14.5 amplicon/primer averages) were obtained from these five bacterial isolates (Supplementary Table S2). Considerable size differentiation from 170 bp (OPW06) to 1720 bp (OPB18) among the amplicons was recorded (Fig. 3). Whereas, polymorphic amplicons (%) from 16.67 − 65.0% (OPC02 - OPW06) with average 53.57% was observed (Supplementary Table S2) and considerable variation in the value of polymorphic information content (PIC) from 0.07 (OPC02) to 0.26 (OPW06) with the mean “0.2” was observed (Supplementary Table S2) while, the variation in Rp value from 1.2 (OPC02) to 7.6 (OPW06) with the mean “4.27” was recorded (Supplementary Table S2) Furthermore, highest resolving power (Rp) values 7.6 and 6.0 for the primers “OPW06” and “OPB18” respectively were observed which significantly caused distinction among the bacterial isolates.

Further, the use of six ISSR primers generated a total of eighty-five amplicons from five bacterial isolates (Supplementary Table S2) and significant discrepancy in the size ranging from 150 bp (UBC807) to 1950 bp (UBC811) was observed (Fig. 4) moreover out of 85 amplicons, 51 (60%) showed polymorphism that were ranging from 2 to 15 (IS10 - UBC807) with the mean “8.5” (bands/primer) and considerable percentage 30 − 85.71% (UBC818 - UBC811) across the primers showing 60% average polymorphism. However, highest PIC (0.33) value with the primer “UBC811” while the two primers “UBC818” and “IS10” demonstrated a lower PIC value “0.11” with a mean 0.2 of all ISSR primers (Supplementary Table S2). The Rp value was ranged from 0.8 (IS10) to 8.0 (UBC807) illustrating the mean value “4.4” (Supplementary Table S2). However, the higher Rp values “6.8” and “8.0” were observed by two ISSR primers “UBC811” and “UBC807” respectively which showed a considerable differentiation between these bacterial isolates. Furthermore, a total of 102 amplicons representing 8 (SRAP-4) to 21 (SRAP-8) amplicons/primer with the mean of “12.75” were observed by the eight SRAP primers. Among these, 68 amplicons demonstrated polymorphism with average amplicon “8.5” per primer (Supplementary Table S2; Fig. 5). These results showed the maximum 85.71% (SRAP-8) polymorphism from the lowest 44.44% (SRAP-7) with average of 66.67% (Supplementary Table S2) and the variation in PIC values from 0.18 (SRAP-7) to 0.34 (SRAP-18) was observed. The variation in Rp value was also observed from 2.4 (SRAP-7) to 10.8 (SRAP-8) whereas primer “SRAP-8” showed the highest Rp value (10.8) which significantly differentiates the bacterial isolates.

#### Coalesced analysis of molecular markers data

In this study, a total of 271 amplicons were generated with RAPD, ISSR and SRAP primers of molecular marker system representing “13.55” as an average of the amplicons for each primer and average polymorphism 60.52% for all primers (Supplementary Table S2). The DNA fragments for the isolate “Pst-4” were relatively higher (196 amplicons) than the isolate “Pst-1”, “Pst-3” (183 amplicons) and Pst-5 (181 amplicons). While, the isolate “Pst-2” was found to have the lower DNA fragments (177 amplicons). More, using combined data analysis, a phylogenetic tree was constructed to understand the genetic relationship of these isolates and using dice coefficient, genetic similarity (GS) from 0.76 (Pst-2 and Pst-5) to 0.83 (Pst-1 and Pst-3) was observed and dendrogram of two main clusters was prepared to represent the grouping of five isolates (Supplementary Fig. S1). In our results, all isolates were effectively characterized with RAPD, ISSR and SRAP markers illustrating the positive or/and negative specification of each marker. However, these three showed 100 unique markers in bacterial isolates that corresponds to 36.9% of the total number of amplicons (Supplementary Table S3). The utmost uniqueness of markers was observed in the isolate “Pst-2” (26 markers) followed by Pst-5 (22 markers), Pst-4 (19 markers) and Pst-1 (17 markers) while the isolate Pst-3 (16 markers) demonstrated lower uniqueness in the markers. All bacterial isolates were successfully characterized by RAPD, ISSR and SRAP markers with the specific positive markers based on their geographical distribution and the level of virulence. Seven specific markers viz., OPB06 _500, 605 bp_, OPW06 _230 bp_, UBC827 _290 bp_ UBC811 _715 bp_, UBC818 _395 bp_ and SRAP-3 _1240 bp_ were found in the isolate “Pst-1” and “Pst-3” that were isolated from El-Minia government and demonstrated low virulence. While five specific markers UBC811 _1015 bp_, SRAP-3 _230, 260 bp_ and SRAP-8 _1, 14 bp_ were observed in Pst-2, Pst-4 and Pst-5 bacterial isolates that were isolated from Assiut government and illustrated higher virulence level. The dendrogram (Supplementary Fig. S1) demonstrated that the markers ISSR, SRAP and RAPD used in this study effectively characterized distinguished these five bacterial isolates. Further, the development of PCR based assay in present study may provide a valuable basis for the molecular identification and characterization of *Pseudomonas syringae* pv. *tomato* and consequently may assist to monitor the prevalence of *P. syringae* pv. *tomato* in these regions for the accurate assessment of disease.

## Discussions

The pathogen *Pseudomonas syringae* pv. *tomato* causing bacterial speck disease of tomato is one of the most prevalent pathogen that has widely been reported throughout the world [37] [38] [39] [40]. The identification of bacterial speck pathogen through precise approaches is important for fundamental studies to assess the genetic polymorphism among the strains for the adequate managements of this disease in agriculture. Recently surveys in the agricultural fields in Egypt showed existence of bacterial speck disease of tomato caused by *Pseudomonas syringae* pv. *tomato* which gain significant consideration as it emerged as one of the most significant disease due to destructive yield losses. In present study, marker-specific molecular approaches i.e., PCR, PCR-RFLP, RAPD, ISSR and SRAP were used which illustrates the genetic relationship and the characterization of five *P. syringae* pv. *tomato* strains that were isolated from various tomato growing regions in the agricultural fields in Egypt.

In our study, primer pair 1406f/23S and 1406f effectively demonstrated the significant genetic disparity among the bacterial isolates of *P. syringae* pv. *tomato*. However, these primers have widely been used for the molecular characterization of this pathogen and documented significant and appropriate results [41] for the genetic classification which strongly support the results of present study. Further, the primer pair “MM5F” and “MM5R” for the amplification of *hrpZ* gene also identifies *P. syringae* pv. *tomato* bacterial strains and these primers has also been used for the depiction of *P. syringae* pv. *tomato* [28] and appropriate findings for the identification of this pathogen were observed which are in the support of our findings.

A study by Fisher and Triplett [28] documented to use a primer pair 16-23 S rDNA in the region of Pst and amplified reproducible fragments of 810 bp for all isolates of *P. syringae* pv. *tomato* and demonstrated the geographical distribution of this pathogen which confer the use of these primers for the accurate geographical distribution of this pathogen. Moreover, the genetic relationship was also studied by amplifying “ITS1” region of *P. syringae* pv. *tomato* by using a primer pair “1406f” and “23S” and the length of amplified products was 800 and 900 bp [42] whereas, the identical length of amplified products was obtained in present study. Thus, using identical primers for the amplification of ITS1 region of *P. syringae* pv. *tomato* provides the resemblance in the length of amplified products but the length of 900 bp product was found pitiable and similar results were also documented in previous study [43] when the primer pair “MM5F” and “MM5R” was used for the identification of 56 *P*. *syringae* pv. *tomato*strains that were isolated from infected tomato fruits and leaves in Tanzania and the length of amplified products was 527 bp for all pathovar specific isolates. This differentiation might be due the geographical distribution and occurrence of hosts and variety. In our study, the results of RFLP-PCR analysis using five endonucleases for 16 S-ITS region and 23 S-rRNA gene showed the divergence in the band pattern for one restriction endonuclease (HaeIII) whereas; no geographical correlation was observed from the origin of bacterial isolates. However, the selection of specific restriction enzyme is considered as the most important factor to distinguish and differentiate the strains [44]. In this study, the results of phylogenetic analysis and the molecular characterization of strains provided an easiness to determine the differences and possibilities of existence of sub-species in these regions. The results of PCR-RFLP analysis of 16 S rDNA and/or “ITS” between 16 and 23 S rDNA of this study were identical to the reported results of genus and sub-species differentiation of *Clavibacter* and close phylogenetic association between the subspecies of *Clavibacter michiganensis* [45, 46]. Comparatively identical correlation of *P. syringae* pv. *tomato* strains was also observed by RFLP and multilocus electrophoresis approaches [42–47] these results strongly support the results of present investigation.

In addition, the ITS region analysis with fourteen endonucleases showed considerable differentiation among 29 strains of *P. syringae pathovars* [48] and higher heterogeneicity in *P. syringae* pv. *tomato* strains were observed. Whereas, phylogenetic relationship were not closely related to tomato pathovars but significant correlation was observed among *P. syringae* pv. *syringae*, *P. syringae* pv. *tomato* and *P. syringae* pv. *maculicola* strains [49]. Moreover, molecular identification by PCR analysis approach using specific primer pair (previously reported) demonstrated no significant delineation between 16 and 23 S rDNA regions of Pst in another strains of *P. syringae* [48]. The detection of Pst strains only by the application of these approaches is complicated and therefore more precise and insightful detection approaches were carried out with other PCR primers in present study which may assist the researchers to identify this bacterial pathogen more effectively and precisely in future.

The results of RFLP analysis sequence for *hrpZ* gene using three endonuclease enzymes (TaqI, HaeIII and HindIII) showed no significant inconsistency in the band pattern of the bacterial strains isolated from Assiut and El-Minia Governorates. Whereas, a close relationship in band pattern of all isolates was observed but only NeaI endonuclease enzyme showed considerable difference among the five isolates. While with the “NeaI” restriction enzyme, 536 bp fragment was digested into two sub-fragments in the isolates Pst-2, Pst-4 and Pst-5 that were obtained from various locations of Assiut Governorate and marked as highly virulent. This fragment was also digested in three fragments from the isolates Pst-1 and Pst-3 that were isolated from two locations of El-Minia Governorate, and these were observed as virulent. It can be considered that the digestion differences of *P. syringae* pv. *tomato* amplicons with restriction enzyme “NeaI” may be utilized to differentiate the isolates depending on the disease severity of prospective geographical distribution.

In this study, these five bacterial isolates characterized by using specific molecular markers viz., ISSR, SRAP and RAPD demonstrated the considerable polymorphism between the isolates and also specify the markers towards each isolate. Moreover, the polymorphic bands percentage (PPB) exhibited by SRAP marker was relatively higher (66.67%) than RAPD (53.57%) and ISSR (60.0%). Further, higher resolving power, marker index and polymorphism information content were found recorded for SRAP marker than RAPD and ISSR (Supplementary Table S2). It has also been reported that the application of various markers can generate specific information which assists the researchers to better understand to distinguish the population diversity and their genetic relationship [50]. Therefore, the combination of different marker-based system will be more valuable to study the genetic diversity [51, 52]. By using molecular markers viz., RAPD, ISSR and SRAP the genetic diversity among the isolates of *Photorhabdus* and *Xenorhabdus* was studied and more precise results were recorded for the characterization the of these bacterial strains because these bacterial strains were accurately distinguished by these markers [53] and these reported results confers the findings of present study. The results of this study demonstrate that the combined application of three different molecular markers can easily distinguish and characterize the *P. syringae* pv. *tomato* isolates more precisely and positively depending upon their virulence and geographical distribution.

## Conclusion

The results of present study suggested that the development of PCR assay for the precise identification and characterization of *P. syringae* pv. *tomato* can used as appropriate tool for early detection of bacterial speck pathogen and consequently will be helpful to monitor the distribution and prevalence of *P. syringae.* pv. *tomato* isolates. This information will be helpful for the selection of molecular markers to understand the genetic differentiation of *P. syringae* isolates. Further, these results provide evidence for the effective and precise use of specific markers for the characterization of bacterial speck pathogen and their virulence in future.

## Electronic supplementary material

Below is the link to the electronic supplementary material.

Supplementary Material 1

Supplementary Material 2

Supplementary Material 3

## Data Availability

Not Applicable. **Statement**: The manuscript has been submitted to a pre-print platform under DOI: 10.21203/rs.3.rs-1707647/v1.

## Declarations

### Human and animal studies

The research did not involve any studies with human participants or animal as experimental model.
