# Supplementary material for: Isolation of Pseudomonas syringae pv. Tomato strains causing bacterial speck disease of tomato and marker-based monitoring for their virulence
Source: Mol Biol Rep. 2023 Apr 19;50(6):4917–30. doi: 10.1007/s11033-023-08302-x (PMC10209279; doi:10.1007/s11033-023-08302-x)
Supplement: Supplementary file 1 — Supplementary Material 1 [file 11033_2023_8302_MOESM1_ESM.docx]

**Table S1.** Morphological and physiological characteristics of the isolated pathogenic bacteria.

| **Tests** | **Bacterial strain 1-5** | |
| --- | --- | --- |
| Shape of cell  Motility  Gram staining  Spore forming  Aerobiosis  Gelatin liquefaction  Asculin production  Potato rot  Nitrate reduction  Starch hydrolysis  Levan production  Indole formation  ***Fermentation of carbon compounds***  Sucrose  Glucose  Fructose  Lactose  Galactose  Raffinose  Mannose  Arabinose | | Rod  +  -  -  +  +  +  -  +  -  +  +  Acid  Acid  Acid  Acid  Acid  -  -  - |

+ = Positive reaction - = Negative reaction

**Table S2.** Summary of RAPD, ISSR and SRAP primer combination

| **Primers** | **TB** | | **PB** | **PPB** | **PIC** | **MI** | **RP** |
| --- | --- | --- | --- | --- | --- | --- | --- |
| ***RAPD primers*** | | |  |  |  |  |  |
| OPB18 | 17 | | 11 | 64.71 | 0.24 | 2.69 | 6 |
| OPA15 | 13 | | 7 | 53.85 | 0.20 | 1.38 | 3.6 |
| OPC02 | 12 | | 2 | 16.67 | 0.07 | 0.13 | 1.2 |
| OPU19 | 7 | | 3 | 42.86 | 0.18 | 0.55 | 2 |
| OPB06 | 15 | | 9 | 60.00 | 0.23 | 2.11 | 5.2 |
| OPW06 | 20 | | 13 | 65.00 | 0.26 | 3.33 | 7.6 |
| Total | 84 | | 45 | -- | -- | -- | -- |
| Average | 14 | | -- | 53.57 | 0.20 | 1.70 | 4.27 |
| ***ISSR primers*** | | | | | | | |
| UBC807 | 24 | | 15 | 62.50 | 0.23 | 3.50 | 8 |
| UBC809 | 13 | | 9 | 69.23 | 0.23 | 2.10 | 4 |
| UBC827 | 18 | | 10 | 55.56 | 0.20 | 2.04 | 5.2 |
| UBC811 | 14 | | 12 | 85.71 | 0.33 | 3.98 | 6.8 |
| UBC818 | 10 | | 3 | 30.00 | 0.11 | 0.34 | 1.6 |
| IS10 | 6 | | 2 | 33.33 | 0.11 | 0.21 | 0.8 |
| Total | 85 | | 51 | -- | -- | -- | -- |
| Average | 14.16 | | -- | 60 | 0.20 | 2.03 | 4.40 |
| ***SRAP primers*** | | | | | | | |
| SRAP-1 | 14 | | 9 | 64.29 | 0.25 | 2.26 | 5.2 |
| SRAP-2 | 17 | | 9 | 52.94 | 0.20 | 1.78 | 4.8 |
| SRAP-3 | 14 | | 9 | 64.29 | 0.26 | 2.37 | 5.6 |
| SRAP-4 | 8 | | 6 | 75.00 | 0.30 | 1.80 | 3.6 |
| SRAP-5 | 10 | | 6 | 60.00 | 0.22 | 1.34 | 3.2 |
| SRAP-6 | 9 | | 7 | 77.78 | 0.28 | 1.99 | 3.6 |
| SRAP-7 | 9 | | 4 | 44.44 | 0.18 | 0.71 | 2.4 |
| SRAP-8 | 21 | | 18 | 85.71 | 0.34 | 6.17 | 10.8 |
| Total | 102 | | 68 | -- | -- | -- | -- |
| Average | 12.75 | | -- | 66.67 | 0.26 | 2.30 | 4.90 |
| **Total** | 271 | | 164 | -- | -- | -- | -- |
| **Average** | 13.55 | | -- | 60.52 | 0.22 | 2.04 | 4.56 |

**TNB:** Total number of bands, **NPB:** number of polymorphic bands, **PPB:** percentage of polymorphic bands, **PIC** polymorphic information content, **MI** marker index, **RP** resolving power.

| **Markers** | **Isolates** | | **Unique positive markers** | **Unique negative markers** |
| --- | --- | --- | --- | --- |
| ***RAPD*** | Pst-1 | 730 bp (OPB18), 750 bp (OPB06) | | 350, 290 bp (OPB18), 815 bp (OPB06) |
|  | Pst-2 | 450, 425 bp (OPB18), 830 bp (OPW06), | | 545, 420, 380 bp (OPB06), |
|  | Pst-3 | 490, 345 bp (OPA15), 645 bp (OPC02), | | 550 bp (OPU19), 720 bp (OPW06) |
|  | Pst-4 | 175 bp (OPB18), | | 810, 715, 510 bp (OPA15), |
|  | Pst-5 | 805 bp (OPB18), 330, 280, 200, 170 bp (OPW06) | | 450 bp (OPW06) |
| ***ISSR*** | Pst-1 | 620, 510, 370 bp (UBC811), 470 bp (UBC818) | | 585 bp, (UBC809), 1700 bp (UBC811) |
|  | Pst-2 | 670 bp (UBC809), 500, 400 bp (UBC827), | | 605, 400bp (UBC809), 550, 520, 370 (UBC827) |
|  | Pst-3 | 1850, 425, 360 bp (UBC807), 350, 210 bp (UBC827), 190 bp (UBC811) | | 510 bp (UBC807), |
|  | Pst-4 | 1560, 710 bp (UBC807), 790 bp (UBC809), 1950, 1930 bp (UBC811), 460 bp (IS10) | |  |
|  | Pst-5 | 725, 590, 440, 190 bp (UBC807), 910, 375 bp (UBC809), | | 455 bp (UBC809), 530 bp (UBC818), 490 bp (IS10) |
| ***SRAP*** | Pst-1 | 450 bp (SRAP-6), 980 bp (SRAP-8) | | 500 bp (SRAP-2), 850, 640, 480 bp (SRAP-8) |
|  | Pst-2 | 800, 650, 530 bp (SRAP-2), 1900, 800 bp (SRAP-6), 365 bp (SRAP-8) | | 470 bp (SRAP-4), 440, 315 bp (SRAP-5), 390 bp (SRAP-6), 540 bp (SRAP-7), 715 bp (SRAP-8) |
|  | Pst-3 | 280 bp (SRAP-2), | | 350 bp (SRAP-1), 480 bp (SRAP-5), 810 bp (SRAP-8) |
|  | Pst-4 | 900, 610, 590, 505 bp (SRAP-1), 600, 510 bp (SRAP-3), 460 bp (SRAP-8) | | 705, 355 bp (SRAP-3), |
|  | Pst-5 | 615 bp (SRAP-4), 650 bp (SRAP-6), | | 300 bp (SRAP-2), 1480 bp (SRAP-4), 545 bp (SRAP-5), 580 bp (SRAP-7), 510 bp (SRAP-8) |

**Table S3.** Unique DNA bands generated by RAPD, ISSR and SRAP markers.
