# Supplementary material for: Isolation of Pseudomonas syringae pv. Tomato strains causing bacterial speck disease of tomato and marker-based monitoring for their virulence
Source: Mol Biol Rep. 2023 Apr 19;50(6):4917–30. doi: 10.1007/s11033-023-08302-x (PMC10209279; doi:10.1007/s11033-023-08302-x)
Supplement: Supplementary file 2 — Supplementary Material 2 [file 11033_2023_8302_MOESM2_ESM.docx]

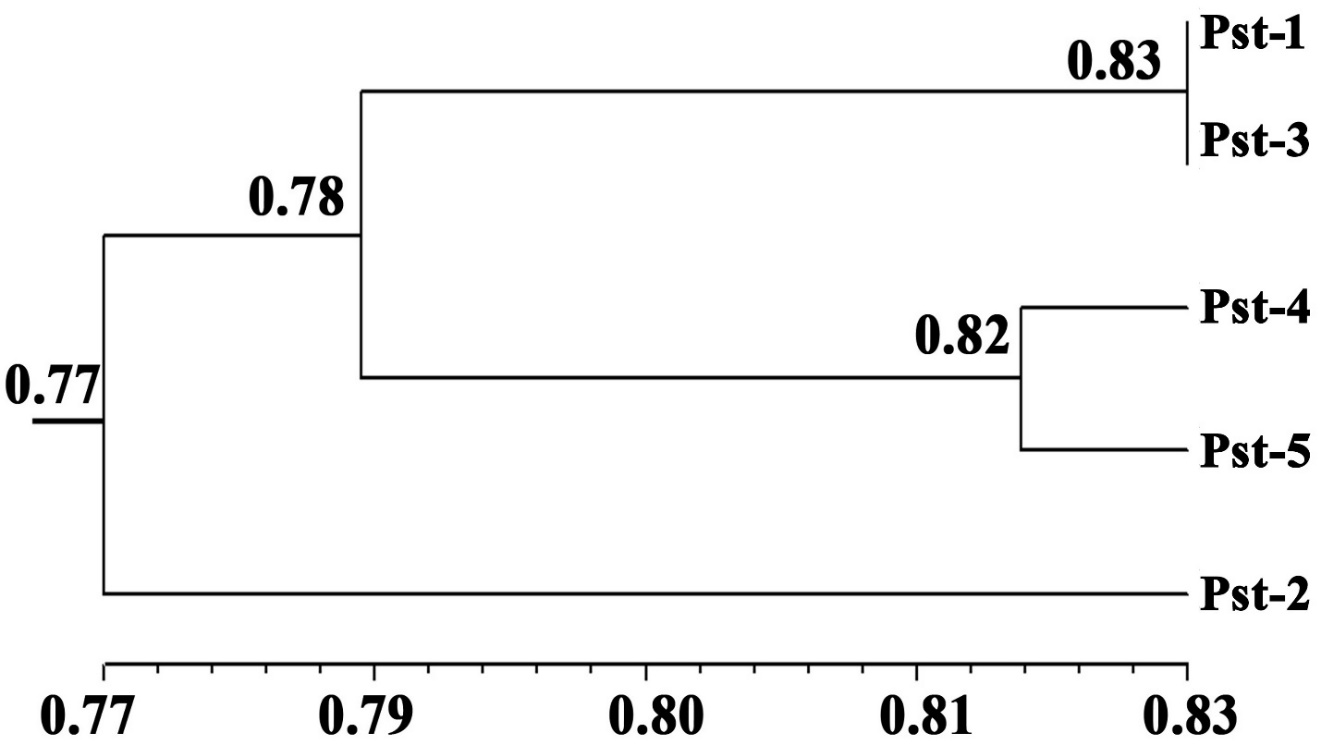


**Figure S1.** A Phylogenetic dendrogram of the relationships among *P. syringae. pv. tomato* five isolates based on UPGMA analysis of the combined data sets obtained with REP-PCR and three molecular marker systems (RAPD, ISSR, SRAP).
