# Supplementary figures and images for: Isolation of Pseudomonas syringae pv. Tomato strains causing bacterial speck disease of tomato and marker-based monitoring for their virulence
Source: Mol Biol Rep. 2023 Apr 19;50(6):4917–30. doi: 10.1007/s11033-023-08302-x (PMC10209279; doi:10.1007/s11033-023-08302-x)

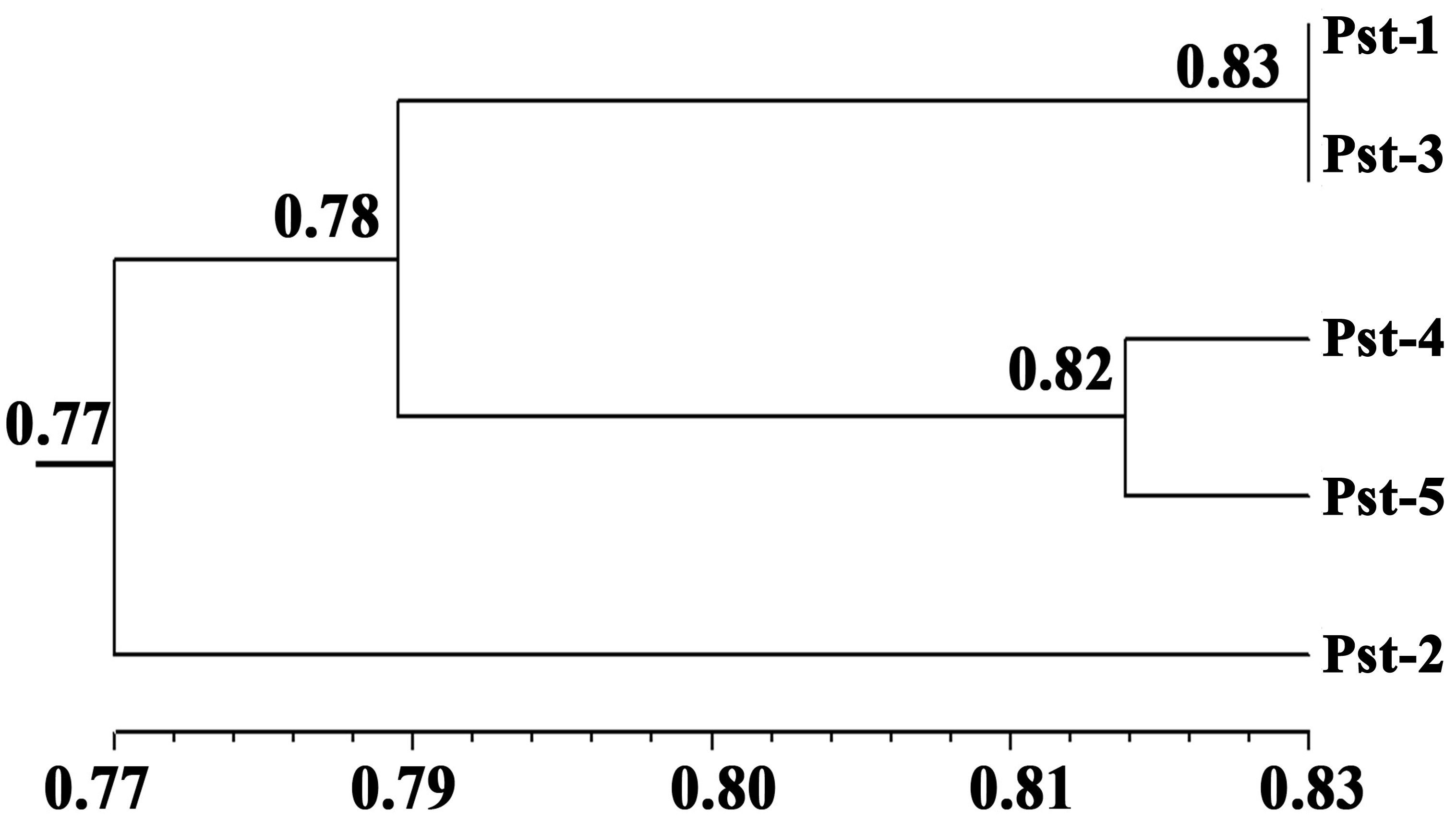

Supplement: Supplementary file 3 — Supplementary Material 3 [file 11033_2023_8302_MOESM3_ESM.jpg]
